# Senior Nursing Officer With Ascites and Abdominal Mass Due to Eosinophilic Gastroenteritis: A Case Report

**DOI:** 10.7759/cureus.83880

**Published:** 2025-05-11

**Authors:** Bingqing Xia, Yu Lin, Qian Feng, Qianyi Liu, Weishan Ruan

**Affiliations:** 1 Gastroenterology, Zhongshan City People’s Hospital, Zhongshan, CHN; 2 Gastroenterology, Southern Medical University Hospital of Integrated Traditional Chinese and Western Medicine, Southern Medical University, Guangzhou, CHN; 3 General Anesthesiology, Zhongshan City People’s Hospital, Zhongshan, CHN

**Keywords:** eosinophilia, eosinophilic ascites, eosinophilic gastroenteritis, laparoscopy, peritoneal disease

## Abstract

Eosinophilic gastroenteritis has non-specific and varied symptoms due to the eosinophil infiltration in various parts of the gastrointestinal system and different wall layers. We reported a 51-year-old female senior nursing officer with dysphagia, vomiting, watery diarrhea, ascites, and abdominal mass. In addition to discussing the difficulties of differential diagnosis, we also face the dilemma of inconsistent treatment opinions between doctors and the patient, especially when the patient is well-informed and resourced.

## Introduction

Eosinophilic gastroenteritis (EG) is a rare, immune-mediated disorder characterized by eosinophil-predominant infiltration of the gastrointestinal (GI) tract. EG exhibits a heterogeneous clinical spectrum, ranging from mild abdominal discomfort to severe complications including obstruction, ascites, or protein-losing enteropathy. The pathogenesis of EG remains incompletely elucidated. Diagnosis requires a combination of clinical symptoms and histopathological evidence of eosinophilic infiltration [1]. Here, we report a case of a 51-year-old female senior nursing officer with EG involving different layers. Despite various symptoms, a previously unreported eosinophil-rich mass was identified during diagnostic exploratory laparotomy. Importantly, beyond history-taking and comprehensive diagnostic evaluation, effective communication with patients, particularly those with a medical background, emerges as a thought-provoking aspect of this case.

## Case presentation

A 51-year-old female senior nursing officer complained of dysphagia without odynophagia, chest pain, acid reflux, heartburn, trachyphonia, weight loss, or choking sensation. She requested an endoscopy in the local hospital and was diagnosed with diffuse esophageal spasm (DES). The symptoms were remitted spontaneously in about a week.

About two weeks later, the patient reported experiencing vomiting and watery diarrhea without fever, chills, hematochezia, or abdominal pain. She self-medicated with montmorillonite (3g PO Tid) for two days. The symptoms were relieved. She assumed it was acute gastroenteritis and did not seek further evaluation. This was the second time that the patient self-medicated.

After one week, she presented with progressive abdominal distention, increased abdominal circumference, and anorexia without any other gastrointestinal, cardiovascular, or genitourinary symptoms, and she was hospitalized. Ultrasound revealed massive ascites with no other signs of pathologic findings. Abdominocentesis obtained slightly turbid, yellow exudatum with ascitic fluid total protein of 38.1 g/L and serum ascites-albumin gradient (SAAG) of 4.2 g/L. The Rivalta test was positive. No evidence of acid-fast bacilli or tumor cells was found. Her purified protein derivative (PPD) came back negative. Symptomatic treatment, including diuretics, was prescribed. Although her symptoms improved dramatically again, she had lost 2 kg within one week, and at her strong request, she was transferred to our hospital for further evaluation. Her blood test results revealed the following: hemoglobin, 136 g/L; white blood cell count, 9.11 × 109/L; and elevated eosinophil count (EO), 3.76 × 109/L. Her medical history was reviewed, and previous results of EO are shown in Table 1.

**Table 1:** Laboratory data of eosinophil count. *Annual physical examination in the local hospital. ●Pre-endoscopy examination in the local hospital. ◆The first blood test after the patient was admitted to the local hospital presenting with progressive abdominal distention. †The last blood test after the patient was admitted to the local hospital presenting with progressive abdominal distention. ★The blood test performed during hospitalization in our hospital.

Time (y-m-d)	WBC (10^9^/L)	EO (10^9^/L)	EO (%)	HGB (g/L)	PLT (10^9^/L)
Normal Range	3.69-9.16	0.02-0.50	0.5-5.0	113-151	101-320
2016-1-8^*^	4.31	0.21	4.9	147	187
2017-5-16^●^	5.49	0.92	16.8	140	204
2017-6-6^*^	11.54	0.76	6.6	134	197
2017-7-9^◆^	10.48	1.22	11.6	155	260
2017-7-18^†^	7.94	2.83	35.6	119	259
2017-7-20^★^	9.11	3.76	41.3	136	262
2017-7-22^★^	8.20	4.20	51.2	126	265
2017-8-3^★^	4.82	1.88	38.0	123	214
2017-8-11^★^	3.63	0.70	19.3	128	215

**Figure 1:**
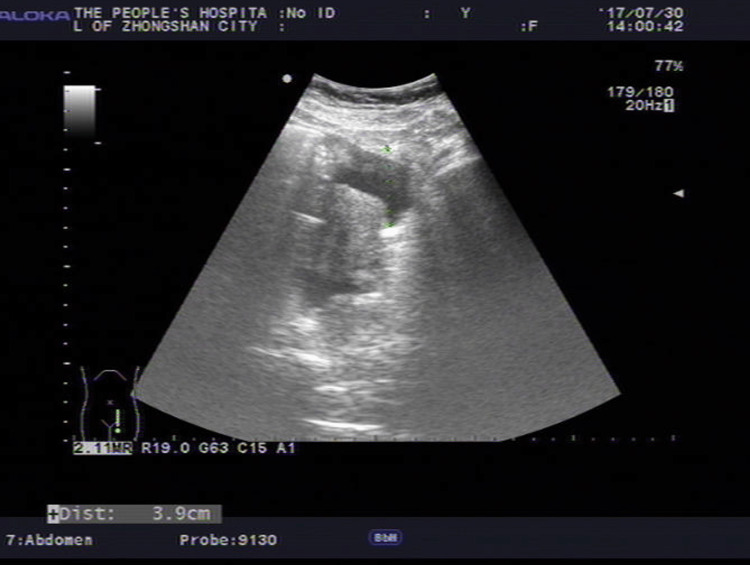
Abdominal ultrasound image of ascites. Ultrasound examination revealed no obvious effusion in the abdomen but a 39 mm fluid sonolucent area in the Douglas pouch.

**Figure 2:**
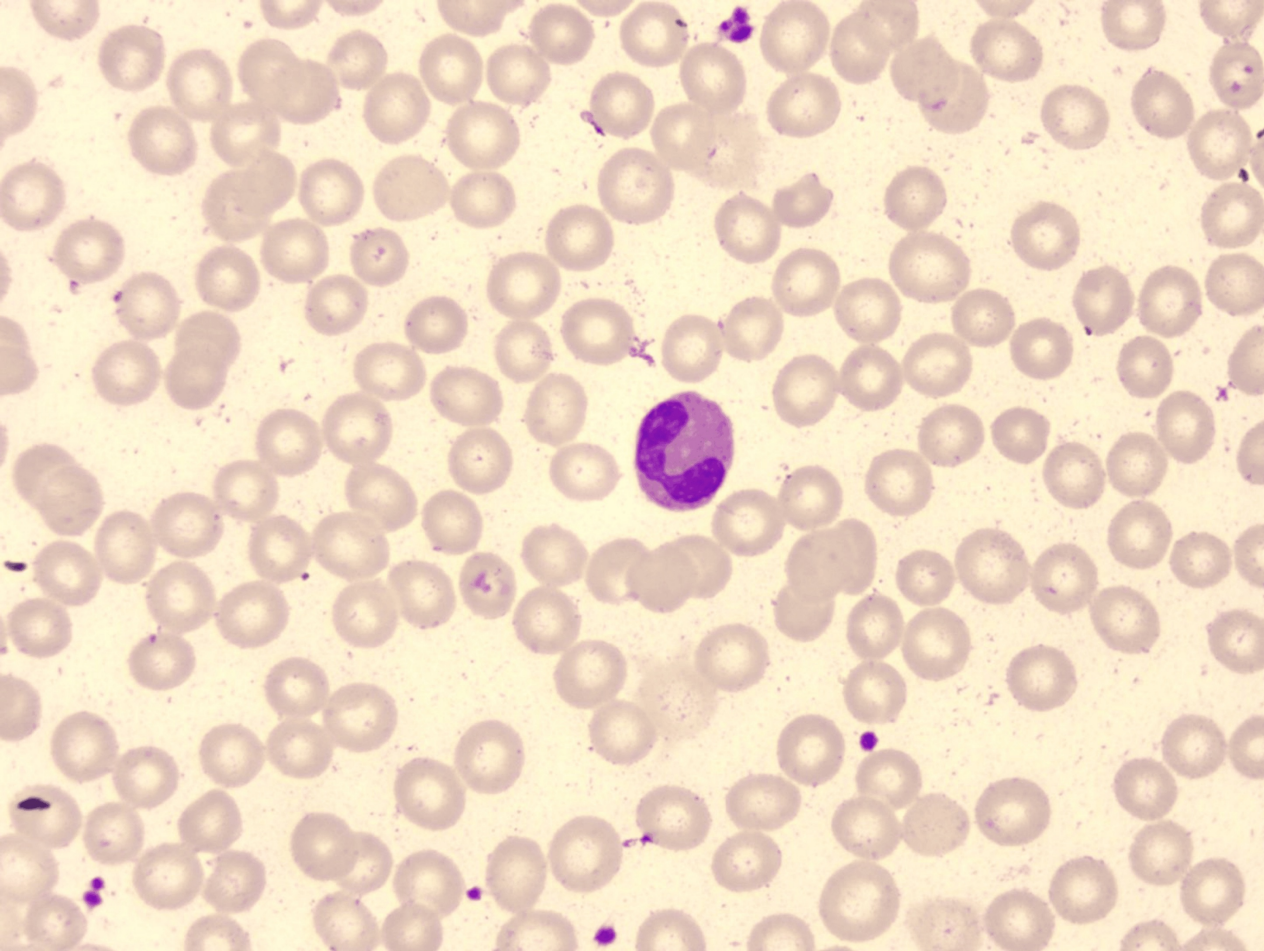
Peripheral blood smears showed the proportion of eosinophils increased. Peripheral blood smears showed that the morphology of granulocytes was normal, but the proportion of eosinophils was increased.

**Figure 3:**
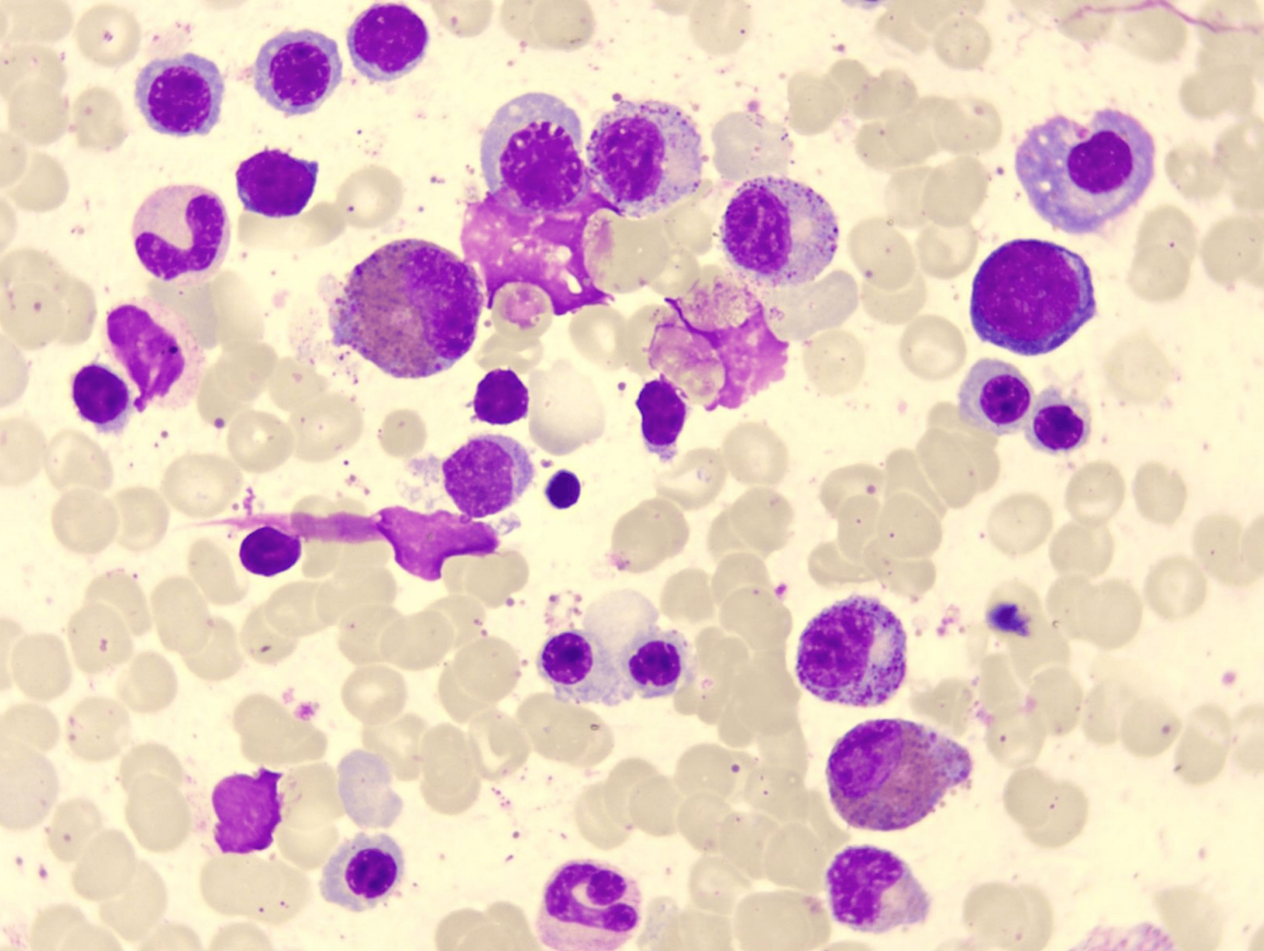
Bone marrow biopsy showed eosinophilic significant aggregates. Bone marrow biopsy showed erythrocytosis, granulocytosis and thrombocytosis while eosinophilic significant aggregates.

**Figure 4:**
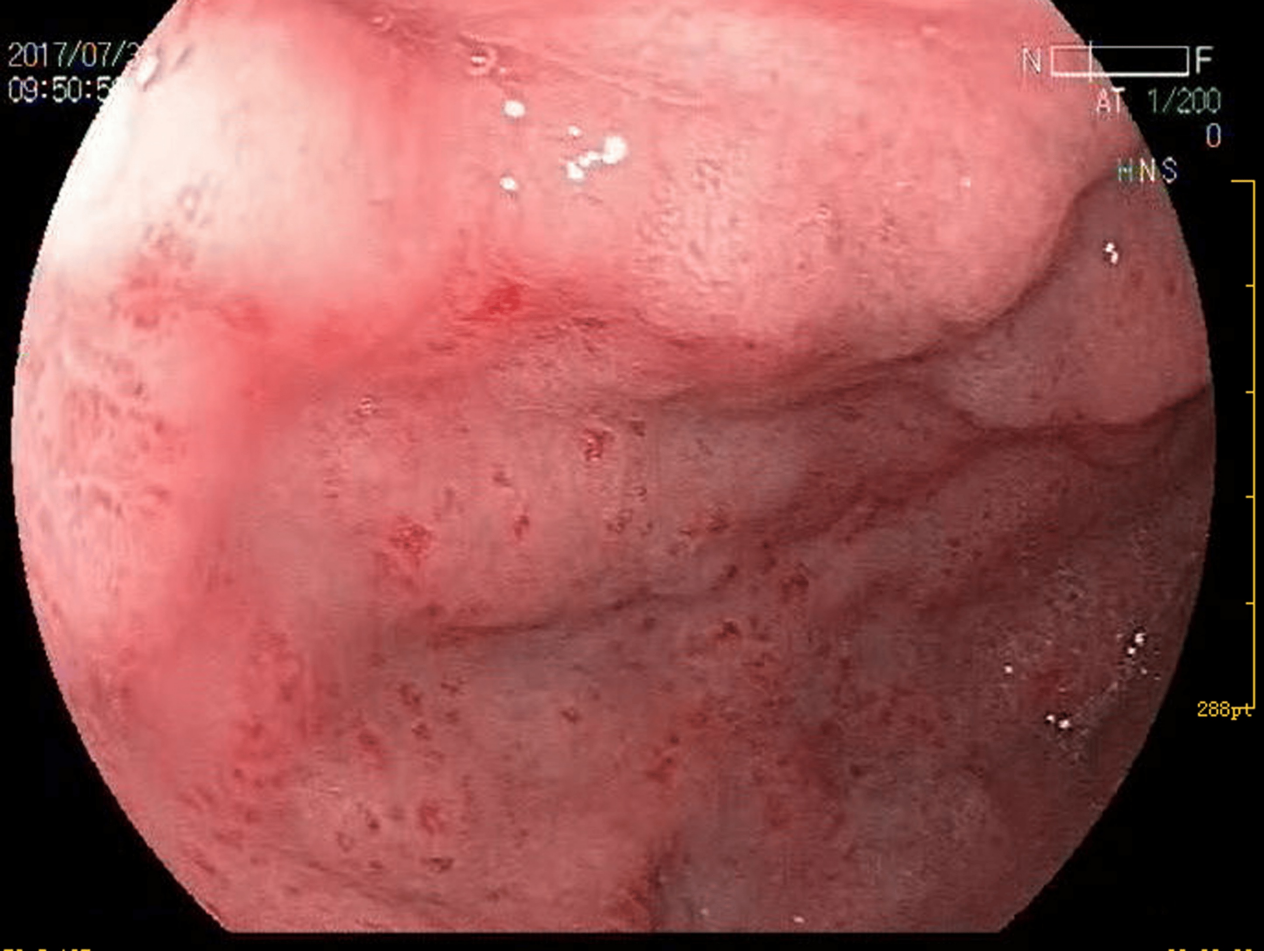
Upper gastrointestinal endoscopy image. Upper gastrointestinal endoscopy showed small patchy congested and erosive lesions in the stomach.

**Figure 5:**
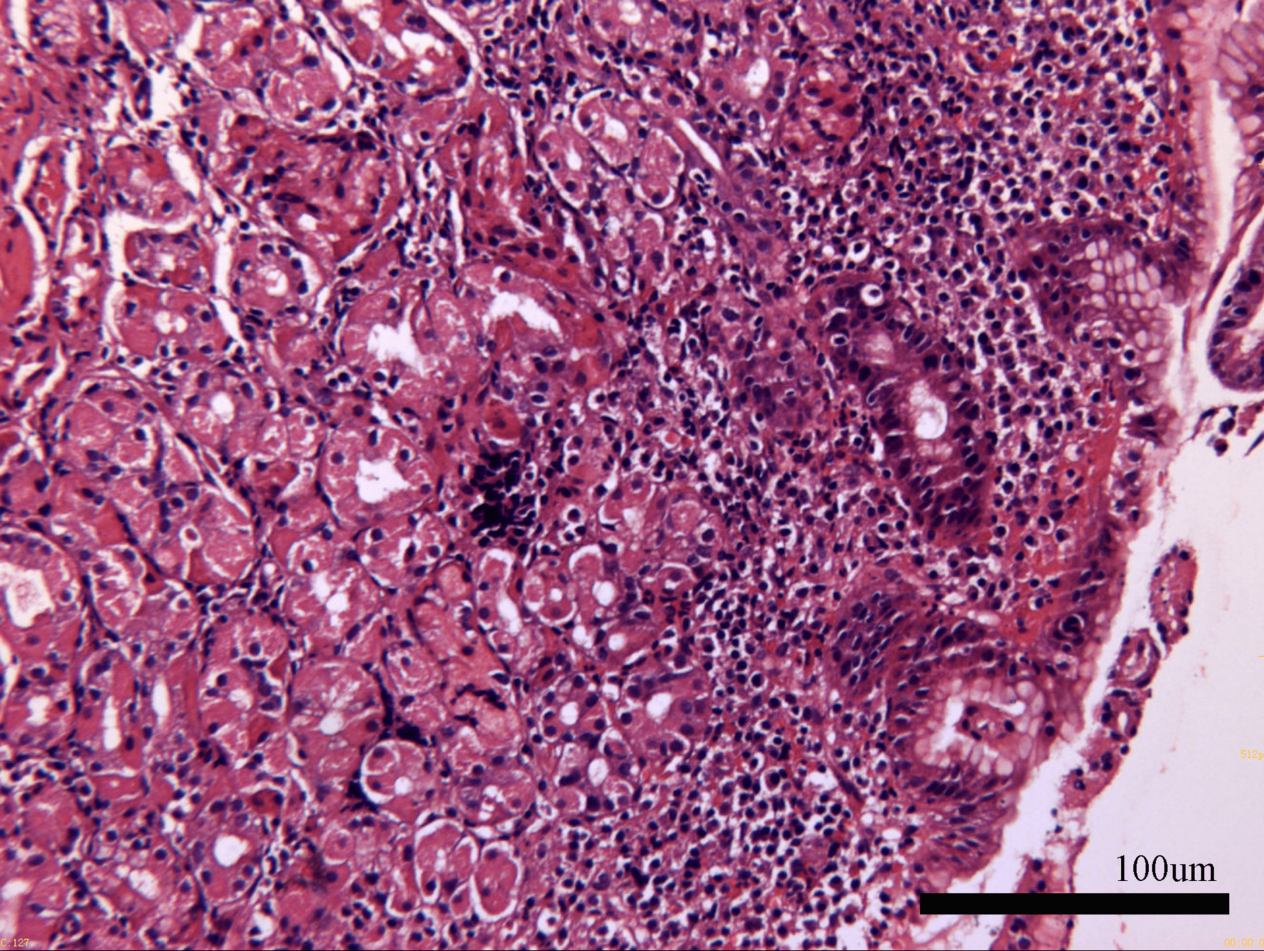
Histopathology image of gastritis biopsy. Histopathology image of gastritis biopsy showed excessive infiltration of plasma cells and lymphocytes.

Her physical examination was normal, and apart from hypoalbuminemia levels (33.9 g/L), blood biochemical examinations showed no obvious abnormalities. Tuberculosis-specific antigen-stimulated interferon gamma release assay (T-SPOT) was negative. Stool test findings revealed no ova parasites, and stool cultures were negative. Diagnostic paracentesis was attempted during the first week after admission but yielded no fluid. Repeated ultrasound examination revealed no obvious effusion in the abdomen, but a fluid sonolucent area with a depth of 39mm was observed in the uterus-rectum-fossa (Figure 1). Before the culdocentesis, intraoperative ultrasound showed no ascites. Peripheral blood smears (Figure 2) and bone marrow biopsy (Figure 3) revealed eosinophilic aggregates with no evidence of myeloproliferative or clonal disorders, and Flow cytometry results were negative. Additional tests showed no multi-organ involvement. Upper gastrointestinal endoscopy showed gastritis with biopsy revealing lymphocytes and plasma cells infiltration in the lamina propria (Figures 4, 5). Colonoscopy identified a proliferative polyp with adenomatous hyperplasia. Pancreatic thin-layer enhanced CT and pelvic enhanced MRI were conducted, both of which showed no significant abnormalities.

**Figure 6:**
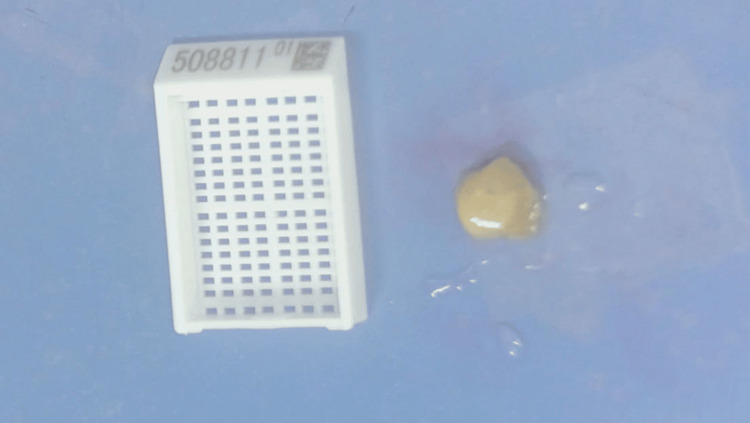
Laparoscope finding. The laparoscope found a 3.0 × 2.0 × 1.0-cm spherical tubercle with moderate hardness at the mesentery root.

**Figure 7:**
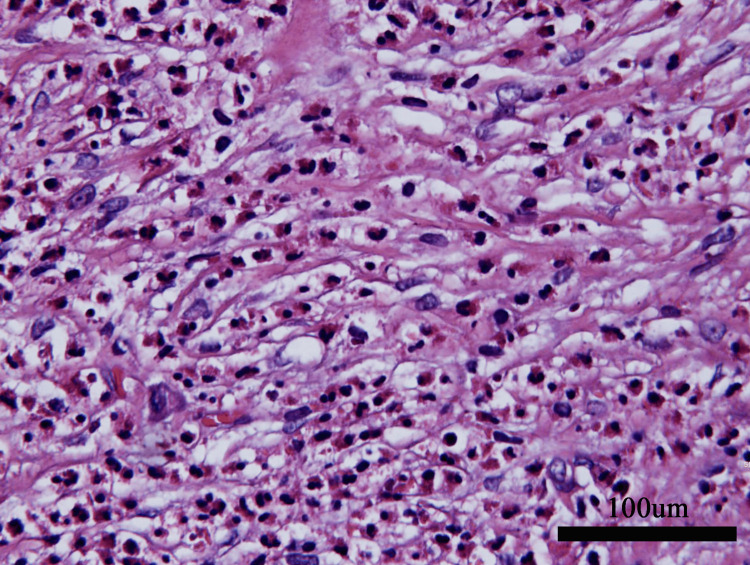
Pathological finding of abdominal mass. Biopsy demonstrated fibrotic tissue containing mature adipocytes and extensive eosinophilic infiltration without granulomas.

The persistent elevated eosinophil levels and family history of pancreatic carcinoma exacerbated her anxiety. After repeated unsuccessful communication attempts, she insisted on undergoing exploratory laparoscopy and a positron emission tomography-computed tomography (PET-CT) scan to ensure comprehensive evaluation. PET-CT showed no significant tumor metabolic imaging. Laparoscopy revealed a 3.0 × 2.0 × 1.0 cm spherical tubercle with moderate hardness at the root of mesentery (Figure 6). Biopsy demonstrated fibrotic tissue containing mature adipocytes and extensive eosinophilic infiltration without granulomas (Figure 7).

She was discharged post-laparoscopy. Her clinical course suggested eosinophilic gastroenteritis (EG), though steroid therapy was withheld due to symptom resolution. Follow-ups at three months and for six subsequent years showed complete remission with normal eosinophil counts and serum albumin.

## Discussion

Notably, EG is a self-limiting, heterogeneous disease of the gastrointestinal tract. Despite its first report in 1937, data on its prevalence are limited because of its rarity, with its estimated prevalence in the USA being 22-28 per 100,000 individuals. The pathophysiology of EG involves eosinophil infiltration in various parts of the gastrointestinal system and different layers of the gastrointestinal wall. Based on the maximum depth of eosinophilic infiltration, the following EG types have been defined: serosal, muscularis, and mucosal types, responsible for 10%, 20%, and 70% of the cases, respectively [1]. Although EG has non-specific and varied symptoms, abdominal pain and nausea/vomiting remain largely consistent across cases. In adolescents and children, EG can present as delayed puberty or growth retardation [2]. Diarrhea, abdominal pain, and/or weight loss are often reported when the primary inflammation location is mucosa. Conversely, the predominant symptoms for muscular layer involvement are nausea, vomiting, and/or abdominal pain. Serosal-type EG presents with eosinophilic ascites, while muscular-type EG presents with symptoms of gastric outlet or small intestine obstruction. Hamamoto et al. reported a rare case of mixed-type EG (mucosal and muscular types) with obstructive jaundice resulting from duodenal wall invagination. Eosinophilic infiltration of the duodenum can cause pancreatic duct obstruction, leading to pancreatitis. Corticosteroid treatment can reverse such obstructions and prevent surgery in most cases.

Importantly, our patient presented with simultaneous involvement of the serosal layer (ascites and mesenteric mass), muscularis layer (dysphagia), and mucosal layer (vomiting and diarrhea).

The first symptom that troubled the patient was dysphagia. The initial differential diagnosis was aimed at identifying whether the dysphagia was esophageal or oropharyngeal. Oropharyngeal dysphagia can cause difficulty initiating swallowing, repetitive swallowing, coughing, choking, and dysarthria. A detailed physical examination and history-taking can provide an accurate assessment in this regard. Considering the characteristics of acute onset, short course, and self-recovery, the first suspects are inflammation, foreign body, or food impaction. Inflammation of the esophageal mucosa may cause congestion or edema, accompanied by symptoms like acid heartburn, reflux, and chest pain. The most common etiologies of esophagitis include eosinophilic esophagitis, reflux esophagitis, infectious esophagitis, and drug-induced esophageal injury. In fact, elevated eosinophil levels had been detected in pre-endoscopy laboratory tests (Table 1), yet were inadvertently overlooked. In such situations, clinicians primarily rely on endoscopy for diagnosis. DES is a relatively rare esophageal motility disorder often characterized by dysphagia and chronic intermittent chest pain. Although it can affect people of all ages, its prevalence is higher in people over 50 years of age and shows no significant gender bias. The lesions typically occur in the lower and middle esophageal segments and manifest as high-amplitude, long-lasting, and non-progressive repetitive contractions. The esophagus may show some spasmodic changes, such as twisting into a spiral shape or transverse false diverticula with no tight wrapping around the endoscopy body at the cardia during endoscopy. Some patients with DES feel relief with the compensatory dilation of the esophagus. If the symptoms relapse, additional tests, such as 24-h esophageal pH impedance monitoring, esophageal manometry, and contrast esophagram, can be considered.

Subsequently, diarrhea appeared. Despite clear evidence of eosinophilic predominance in routine monitoring (Table 1), no targeted interventions were initiated, raising alarms about cognitive biases in autonomous clinical practice.

This previously healthy 51-year-old female medical staff member eventually developed ascites. Notably, the SAAG value plays a critical role in categorizing ascites. A SAAG value of ≥11 g/L indicates portal hypertension. The differential diagnosis includes fulminant hepatic failure, cardiac ascites, acute fatty liver of pregnancy, alcoholic hepatitis, Budd-Chiari syndrome (BCS), hepatocellular carcinoma, myxedema, cirrhosis, massive liver metastases, veno-occlusive disease, and portal vein thrombosis. In contrast, a low SAAG (<11g/L) indicates peritoneal abnormalities. For patients with low SAAG, the following differential diagnoses should be considered: bowel perforation or infarction, peritonitis from connective tissue diseases, peritoneal carcinomatosis, chlamydial peritonitis, tuberculous peritonitis, postoperative iatrogenic lymphatic leakage, pancreatic ascites, and biliary ascites.

Our patient’s low SAAG narrowed the differential diagnoses to peritoneal abnormality-related causes. With an EO far exceeding 1.5 × 109/L, hypereosinophilia (HE) was confirmed. HE can cause spontaneously regressive ascites. Although the diagnosis seemed apparent, several aspects required more evaluation: (1) Beyond eosinophilia, what other diseases could explain the symptoms? Given comprehensive diagnostic evaluations, are additional tests needed? (2) The most notable symptom, rapidly resolving ascites-raised questions about follow-up management: Was conservative observation acceptable?

Peritoneal carcinomatosis is the leading cause of non-hepatic ascites. Our patient’s family history (her father died of advanced pancreatic cancer) necessitated cancer screening. Additionally, in all cases of unknown ascites, rheumatic immune responses must be considered, given their higher prevalence in women. Reports of sporadic cases involving BCS, Janus kinase (JAK2) mutations, and idiopathic eosinophilia exist [3]. However, BCS is inconsistent with the low SAAG parameters, whereas nephritic ascites aligns with this category. Kimura’s disease (KD), characterized by lymphadenopathy, subcutaneous masses, elevated IgE, and peripheral eosinophilia, is strongly associated with renal involvement and predominantly affects Asians [4]. Yu et al. reported KD with nephrotic syndrome, ascites, and eosinophilia, though these are typically considered distinct entities [5]. Noel et al. described eosinophilic pancreatitis with ascites and a homozygous serine protease inhibitor Kazal type 1 (SPINK1) mutation [6]. A study comparing chronic pancreatitis (CP) patients with (n=28) and without eosinophilia (n=152) found higher rates of pancreatic enlargement, jaundice, and pancreatic ascites in the former [7]. Eosinophilic peritonitis generally occurs within three months of initiating peritoneal dialysis [8], with a self-limiting course. However, tuberculosis-associated eosinophilic peritonitis during peritoneal dialysis has been reported [9].

Our patient lacked alarm symptoms beyond weight loss, which itself was unreliable due to ascites resolution. Although occult malignancy cannot be fully excluded, no evidence supports malignancy or rheumatic disease (normal inflammatory markers, absence of rash, alopecia, arthralgia, or autoantibodies). Other diagnoses were ruled out through detailed history and targeted investigations.

Eosinophilic ascites is the hallmark feature of serosal involvement and should be considered in cases of unexplained ascites presenting with gastrointestinal symptoms. Given that diagnostic delay can prolong patient suffering, a high index of suspicion is essential. The laparoscope did help reveal a rare manifestation of EG. In a study describing the laparoscopic features of two patients with serosal-type EG [10], hyperemia of the peritoneal serosa was noted in one case, while the other showed parietal peritoneal hyperemia, thickening, and multiple scattered irregular whitish-yellow nodules. In our patient, laparoscopy revealed a spherical tubercle with moderate hardness at the mesenteric root. To our knowledge, this is the first report of EG presenting with full-layer involvement and a mesenteric mass. Interestingly, Pineton de Chambrun et al. [11] noted that predominantly serosal-type EG has a favorable prognosis, often resolving after a single flare without chronic progression. Conversely, mucosal-type EG tends to follow a chronic course with higher recurrence rates.

While exploratory laparoscopy enhanced our understanding of EG in this case, its necessity remains debatable. Laparoscopy provides visual access to the peritoneal cavity while facilitating targeted biopsies for microbiological and histological studies. Some EG had been diagnosed only after resecting the obstructing segment after laparotomy [12] or biopsy of the distal ileum [13]. A rare case of predominantly subserosal-type EG accompanied by colon cancer has been reported. However, the relationship between colon cancer and EG remained unclear. These operations had clear indications, but not all procedures yield clinical benefits. A patient had partial lipodystrophy, pancreatitis, and recurrent eosinophilia and underwent five laparotomies. On the sixth operation, the patient died of uncontrollable, fatal hemorrhage [14]. This case highlighted risks in over-investigation for a condition like EG, which typically has a benign course.

## Conclusions

When doctor-patient disagreements arise, balancing patient autonomy with medical rigor is challenging. In China’s strained healthcare climate, clinicians face unprecedented scrutiny, and transferring excessive decision-making authority to patients may reflect defensive medicine. This issue intensifies when patients are medically literate and resourceful. Medical professionals often self-manage care due to their healthcare expertise, but this risks over-treatment, escalating costs, and eroding public trust. Our case underscores the importance of objective clinical synthesis and adherence to standard guidelines. Healthcare workers must avoid self-diagnosis and collaborate with treating physicians to ensure balanced care.
